# Development of language-specific stress discrimination in European Portuguese: an electrophysiological study

**DOI:** 10.3389/fnins.2024.1415854

**Published:** 2024-09-20

**Authors:** Shuang Lu, Cátia Severino, Marina Vigário, Sónia Frota

**Affiliations:** ^1^School of Foreign Languages, Renmin University of China, Beijing, China; ^2^Center of Linguistics, School of Arts and Humanities, University of Lisbon, Lisbon, Portugal

**Keywords:** infant stress perception, European Portuguese, ERP, mismatch response, iambic advantage

## Abstract

European Portuguese (EP) is a language with unpredictable stress. Previous behavioral studies have shown that without vowel reduction EP adult speakers displayed a stress deafness effect akin to that observed in speakers of fixed-stress languages, suggesting that vowel quality may be the primary cue for stress discrimination in EP. However, an event-related potentials (ERPs) study reported that EP adults were able to discriminate stress contrasts pre-attentively in the absence of vowel quality cues. These results seemed to indicate that EP adult speakers may attend to different cues in the attentive and pre-attentive stress perception. Moreover, both the behavioral and ERPs studies have revealed a processing advantage for iambic stress, which could not be predicted by the rhythmic properties of EP, the language-specific weighting of stress correlates, or the frequency distributions of trochaic and iambic stresses in EP. A recent eye-tracking study has found that EP-learning infants at 5–6 months already exhibited an iambic preference in the absence of vowel reduction, manifested by longer looking time at the iambic stress. The present study used a passive oddball paradigm to examine pre-attentive stress perception without vowel quality cues by 5-to-7-month-old EP-learning infants. Results from twenty-two participants showed that both the trochaic and iambic conditions yielded a positive discrimination response (p-MMR). In addition, the iambic condition elicited a prominent late discriminative negativity (LDN) as well as a P3a component. Our findings present the first evidence for reciprocal discrimination of stress patterns in EP-learning infants, showing that, as in adult speakers, stress processing might also differ at the pre-attentive and attentive stages in infants. Importantly, the stress perception ability in EP-learning infants seems to develop asymmetrically, with an advantage for the iambic stress pattern. The present study highlighted the role of language-specific factors that may affect developing stress perception.

## 1 Introduction

The perception of prosody plays a crucial role in infants’ language acquisition. The prosodic bootstrapping hypothesis argues that infants use prosodic features, such as word stress and intonational phrasal boundaries, to learn the lexical and morphosyntactic information of their native languages (e.g., Wanner and Gleitman, 1982; Höhle, 2009; Gervain et al., 2020, for a recent review). Previous empirical research has found that infants’ stress discrimination abilities emerge at birth (Sansavini et al., 1997), and develop in language-specific ways between 4 and 9 months of age (e.g., Becker et al., 2018; Friederici et al., 2007; Jusczyk et al., 1993). This early sensitivity to lexical stress may facilitate infants’ word segmentation and thus word learning (Jusczyk et al., 1999; Nazzi et al., 2006; Shi et al., 2006; Shukla et al., 2011; Polka and Sundara, 2012). Stress perception has also been regarded as an early indicator of infants’ later language development (Friedrich et al., 2009; Weber et al., 2005). This article presents the first ERP study on the development of stress processing in European Portuguese, a language where the cues to word stress offer a challenging combination of features hitherto not examined in the ERP literature on developing stress perception.

Languages vary in a number of aspects regarding word stress, such as stress position and the acoustic correlates of stress. In some languages, the placement of stress is fixed (e.g., Finnish, Hungarian, and Polish), while in others the position of stress is mostly unpredictable (e.g., English, Spanish, and Russian), and this unpredictable stress can create minimal pairs, such as /'InsaIt/ *insight* versus /In'saIt/ *incite*. Since stress processing is particularly important in languages with variable stress position, infants who are learning these languages demonstrated better stress discrimination abilities compared with French-learning monolingual infants who do not use contrastive stress lexically in their native language (e.g., Abboub et al., 2015; Bijeljac-Babic et al., 2012; Friederici et al., 2007; Höhle et al., 2009; Skoruppa et al., 2009, 2011, 2013). This has been found in contexts with limited segmental variability (using a disyllabic item produced with either a trochaic or an iambic stress), as well as in contexts with segmental variability (using segmentally varied trochaic and iambic disyllables).

In addition, lexical stress is signaled by different acoustic cues across languages. It is widely accepted that all acoustic cues are examined simultaneously when determining lexical stress in English, and that none of them, such as pitch, duration, or intensity *per se*, is the single cue (Liberman, 1960). But in some languages, such as Polish and Thai, stress may be marked uniquely by one acoustic cue (Dogil and Williams, 1999; Potisuk et al., 1996). Even when the same acoustic correlates of stress are employed, languages may differ in the exact weightings for the same cues (Bleakley, 1973; Llisterri et al., 2003). A previous study on Italian-learning infants has suggested that the pitch and duration weighting may follow separate developmental paths, with the weighting of duration emerging from language experience (Bion et al., 2011). Bion et al. (2011) also pointed out that the early prosodic bias may be highly influenced by the acoustic cues in the speech stream. A trochaic bias may be elicited by prominence marked by pitch, while an iambic bias may be triggered by prominence marked by duration. This claim has been partially supported by empirical results obtained from English-learning infants and Hebrew-learning infants. In English, relative pitch prominence is considered as the primary cue for stress (e.g., Fry, 1958; Morton and Jassem, 1965), while in Hebrew duration is the main acoustic correlate for stress (e.g., Bat-El et al., 2019; Most, 1999). In the presence of segmental variability, English-learning 9-months-old infants have been found to exhibit a trochaic preference (Jusczyk et al., 1993), whereas Hebrew-learning 9-month-olds prefer iambs over trochees (Segal and Kishon-Rabin, 2012). However, studies on German-, Spanish-, and Catalan-learning infants presented conflicting evidence on the link between the early prosodic bias and the dominant acoustic cues in the speech stream. Even though duration is regarded as the most reliable cue to word stress in German (e.g., Dogil and Williams, 1999; van der Hulst, 1999; Jessen et al., 1995), German-learning infants displayed a trochaic advantage as early as 4–6 months of age (Höhle et al., 2009; Weber et al., 2004). Moreover, duration has been found to be the primary cue to stress in both Spanish and Catalan (Astruc and Prieto, 2006; Ortega-Llebaria et al., 2008, 2010), but neither Spanish-learning nor Catalan-learning infants have demonstrated a preference for either stress pattern at 6 or 9 months of age (Pons and Bosch, 2007). These results suggest that the weighting of stress cues alone could not explain infants’ preference for a specific lexical stress pattern.

Another possible explanation for infants’ asymmetrical processing of stress is the rhythmic properties of the language. According to the rhythmic-activation proposal, infants’ early word segmentation is guided by the rhythmic unit of their native language. Stress preference should only be evident when infants are learning stress-based languages such as English, Dutch, or German, yet should not emerge for infants learning syllable-based languages such as Catalan, French, Italian, or Spanish. Nazzi et al. (2006) showed that English-learning infants relied on a trochaic stress unit to segment words, while French-learning infants used a syllabic strategy instead. This could explain the absence of stress preference in both the Spanish- and Catalan-learning infants.

Furthermore, it might be the frequency of stress patterns in the language that trigger the iambic or the trochaic bias. For example, the predominant pattern in English and German is trochaic stress (e.g., Alber, 2020; Cutler and Carter, 1987; Vennemann, 1990), but iambic stress predominates in Hebrew (Segal et al., 2009; Segal and Kishon-Rabin, 2012). By contrast, in Spanish and Catalan the difference between the frequencies of the two stress patterns is smaller (Pons and Bosch, 2007). Nevertheless, Pons and Bosch (2010) found that Spanish-learning infants’ stress preferences might be modulated by word shape. According to the LEXESP database (Sebastián-Gallés et al., 2000), 95% of the Spanish CVC.CV words have trochaic stress, whereas 93% of the CV.CVC words have iambic stress. Although 9-month-old Spanish-learning infants did not show a stress preference on CV.CV pseudo-words, a trochaic and an iambic preference was observed when they were tested with CVC.CV and CV.CVC items respectively (Pons and Bosch, 2010).

Previous research seems to suggest that the rhythmic properties of the native language might determine whether infants may develop a stress preference, and the language-specific weighting of stress correlates along with stress frequency distribution influence which stress pattern infants may prefer. However, European Portuguese (EP) is a typologically interesting language that differs from English, German, French, Spanish or Catalan regarding its rhythmic properties and stress correlates, and from English and Hebrew regarding the frequency distributions of stress patterns (Frota et al., 2020, for a recent review of prosodic features and lexical stress in EP). EP has a prosodic profile that includes mixed features of stress-timed and syllable-timed languages (Frota and Vigário, 2001). The language displays contradictory frequency distributions of trochaic and iambic stress, with different stress patterns predominating if type or token frequency is considered, and if frequency is computed over the lexicon or at the prosodic word level in connected speech (Vigário et al., 2010; Frota et al., 2020). Additionally, duration and vowel quality, rather than pitch, have been found to be the primary perceptual correlates for stress in EP. Therefore, investigating stress perception in EP-learning infants can broaden the understanding of the factors that influence the development of infants’ early stress perception.

EP has variable stress, which may fall on one of the last three syllables of a prosodic word. Although the language has mixed rhythm, Frota et al. (2002) showed that the prosodic perception of native EP adult speakers relied more on syllable-timed properties, as they discriminated EP from Dutch based solely on prosodic cues. This result pointed to a syllable-timed nature of EP and suggested that EP-learning infants might segment speech similarly to infants who are learning a syllable-timed language. However, Butler and Frota (2018) revealed that unlike Spanish-, Catalan-, or French-learning infants, who are learners of syllable-timed languages, EP-learning infants did not use the syllable in a similar way as the major rhythmic unit for segmentation, probably due to the mixed rhythmic properties of EP. Hence, based on the rhythmic properties alone, we are unable to predict whether EP-learning infants would develop an early processing advantage for a given stress pattern.

According to the FrePOP and P-PAL databases (Frota et al., 2010; Soares et al., 2018), penultimate stress is the predominant pattern in EP. More than 65% of disyllabic words (the most frequent word type) in adult speech have trochaic stress. A rather similar distribution has also been found in child-directed speech (Vigário et al., 2006). However, iambic stress becomes slightly more frequent than trochaic stress if monosyllabic stressed words are taken into account (Vigário et al., 2010). We should consider the monosyllabic stressed words to have iambic stress for two fundamental reasons. First, it was reported by Vigário et al. (2006) that EP monosyllabic words and stress-final syllables share a number of properties. Second, clitics make up around 30% of all word tokens in EP (Frota et al., 2006; Viana et al., 1996). A great majority of these clitics are unstressed syllables that attach to the following stressed word, yielding an iambic stress pattern. Thus, we cannot draw a definitive conclusion from the frequency of stress patterns in EP regarding which stress pattern predominates.

With regard to the correlates of stress, vowel quality has been reported to be the main cue for stress perception in EP. Prior behavioral studies have shown that without vowel reduction EP adult speakers displayed a stress “deafness” effect (also referred to as “stress insensitivity”, see Nikolić and Winters, 2022), which is comparable to what was observed in speakers of languages with no lexical stress or fixed stress (Correia et al., 2015; Lu et al., 2018). When the vowel quality cue is not present, duration has been found to be the primary cue for word stress in EP (Andrade and Viana, 1989; Delgado-Martins, 1977). However, pitch is not considered as a correlate of word stress, because most stressed syllables in EP lack a pitch accent (Frota et al., 2002; Frota, 2014). In a previous ERP study, EP adult speakers demonstrated discrimination between trochaic and iambic stress in the absence of vowel quality cues at the pre-attentive stage, suggesting that stress processing might differ at the pre-attentive and attentive stages, with duration emerging as a sufficient cue for stress only in the former (Lu et al., 2018). Moreover, both behavioral and ERP studies demonstrated a processing advantage for iambic stress in adult speakers’ stress perception (Lu et al., 2018). Using an anticipatory eye movement paradigm, Frota et al. (2020) conducted the only study on EP-learning infants’ perception of stress. They found that 5–6 month-old EP-learning infants displayed an iambic preference. Specifically, in the presence of segmental variability and in the absence of vowel quality cues, infants looked longer at disyllabic pseudo-words with iambic stress than at those with trochaic stress. This finding provided evidence for the early development of asymmetrical perception of iambic stress in EP.

Previous behavioral and ERP studies on native Russian speakers have shown conflicting results concerning the asymmetrical processing of stress patterns. Russian, unlike EP, is a stress-timed language; however, and like EP, it has a controversial frequency distribution of trochaic and iambic stress patterns (e.g., Mitciuk and Pelts, 2022). Using ERP measures, Molczanow et al. (2013) demonstrated that trochaic stress is less costly in prosodic processing than iambic stress, whereas Crosswhite et al. (2003) found evidence in favor of an iambic advantage using behavioral measures.

To our knowledge, no ERP study has been conducted on EP-learning infants to examine whether they would show stress discrimination and/or asymmetrical stress perception at the pre-attentive stage. It is thus unknown whether EP-learning infants already show adult-like stress discrimination (as reported in Lu et al., 2018), and whether they exhibit a language-specific asymmetrical stress perception (as reported in Frota et al., 2020). Therefore, the present study adopted a passive oddball paradigm to record ERPs from 5 to 7-month-old EP-learning infants, in order to investigate whether they already developed a stress discrimination ability and asymmetrical stress perception inattentively, in the absence of vowel reduction.

We mainly focused on three ERP components which have been extensively used to study auditory discrimination. The mismatch negativity (MMN) is a frontocentrally-distributed negative wave elicited by an infrequent change in a sequence of standard stimuli. It typically peaks at 100–300 milliseconds after change onset in adults, but may vary slightly depending on different paradigms and the type of deviant stimuli (e.g., Näätänen et al., 1993, 2004). The MMN has also been found in infancy, but with a positive polarity (i.e., positive mismatch responses, p-MMR, e.g., Leppänen et al., 1997; Morr et al., 2002; Weber et al., 2004). Research has suggested that the p-MMR matures towards the adult-like MMN between 3 and 9 months of age, despite a wide individual variation (e.g., Jing and Benasich, 2006; Maurer et al., 2003; Trainor et al., 2003). Besides neural development, stimulus parameters (e.g., Cheng et al., 2015) and signal processing approaches, such as filter setting (e.g., Trainor et al., 2003; Weber et al., 2004), may also influence the polarity of the mismatch response. Moreover, in infants and young children, the peak latency and the scalp distribution of the mismatch response tend to be longer and broader than that in adults (e.g., Cheour et al., 1998; Cheour-Luhtanen et al., 1995, 1996). A prominent mismatch response may not only be observed over the frontal and central areas but also over the parietal area in infants. The second component that can be elicited by deviant stimuli in a passive oddball experiment is late discriminative negativity (LDN). The LDN is also a frontally dominant negativity which follows MMN and peaks around 300–600 milliseconds after the change onset (e.g., Korpilahti et al., 2001). Some studies have suggested that the occurrence of LDN may be language-specific (e.g., Yu et al., 2017), and it may represent auditory rule extraction (e.g., Zachau et al., 2005), or involuntary reorientation of attention (e.g., Escera et al., 2000; Shestakova et al., 2003). Other studies have argued that an increase in LDN amplitude may reflect increased automatic detection of phonological representations (e.g., Alonso-Búa et al., 2006), and the absence of the LDN may suggest reduced ability of auditory discriminative processing (e.g., Azaiez et al., 2023; Cheour et al., 2001). Lu et al. (2018) reported that for adult EP speakers the MMN and the LDN components in the iambic condition were more negative and extended across a larger temporal window than that in the trochaic condition. These two components were also accompanied by EP adults’ better performance on iambic trials in a behavioral task, indicating a processing advantage for iambic stress at the pre-attentive and attentive stages in adult EP speakers. In the present study, if EP-learning infants also demonstrated asymmetrical stress perception at the pre-attentively stage, we expect to find larger MMN and LDN amplitudes for iambic stress than for trochaic stress. Another ERP component that often follows the MMN is the P3a, which has been argued to index rapid involuntary attention switching (e.g., Escera et al., 1998; Polich, 2007). This positive component typically emerges between 300 and 400 milliseconds after deviant stimulus presentation, but may extend to 900 milliseconds (Patel and Azzam, 2005). P3a differs from the P300 or P3b component in that it has shorter latency and more frontally-oriented topography (Knight et al., 1989; Squires et al., 1975). Previous research has found that the larger the acoustic difference between the deviant and standard sounds, the larger the P3a component (e.g., Escera and Corral, 2007; Friedman et al., 2001). Besides, P3a elicitation may only happen when preceded by a significant MMN response (e.g., Escera et al., 1998; Novitski et al., 2004).

In summary, the current study used the ERPs method to examine early stress perception abilities in the absence of vowel reduction in 5–7-month EP-learning infants. Previous behavioral and ERP studies have shown that language-specific asymmetrical perception of stress patterns emerges after 4 months of age in some languages (e.g., Höhle et al., 2009; Weber et al., 2004). Since EP is a language that has a mixed prosodic profile and controversial stress frequency distributions, combining a diverse set of prosodic and segmental cues to stress, it is difficult to predict whether EP-learning infants would demonstrate stress discrimination and a language-specific asymmetrical stress perception pre-attentively. A previous study using the eye-tracking method has shown that 5–6-month-old EP-learning infants exhibited a preference for iambic stress (Frota et al., 2020). However, given that conflicting evidence for stress discrimination was found for adult EP speakers, stress processing might also differ at the pre-attentive and attentive stages in infants. Using infants at similar ages as in Frota et al. (2020), the present study aims to test whether EP-learning infants would demonstrate stress discrimination, and if so if they equally discriminate trochaic and iambic stress or exhibit an iambic advantage at the pre-attentive stage.

## 2 Materials and methods

### 2.1 Participants

Using G*Power version 3.1.9.7 (Faul et al., 2007), we performed an a priori power analysis to determine the minimum sample size for our study. Results showed that to detect a medium effect (*f* = 0.25) at a significance criterion of α = 0.05 the required sample size to obtain .80 power was 19 for 2 × 2 × 2 repeated measure ANOVAs. The data from twenty-two infants (13 females), who were raised in monolingual EP families from the wider Lisbon area, was included in the analysis. The age range of the infants was between 5 months 7 days to 7 months 11 days (M = 6 months 18 days, SD = 17 days). The infants were recruited from the Lisbon Baby Lab database of participants, and parents were given a complimentary voucher in appreciation for their participation in the study. For all the infants tested, parents reported no health-related issues (including hearing-related problems), and no familial risks for language impairment. According to an EP adapted version of the Communication and Symbolic Behavior Scales Developmental Profile (CSBS DP) Checklist, a widely used tool for the screening of early language and social communication skills (Filipe et al., 2023), the participants were typically developing infants with scores as expected for their age range (Table 1). An additional nineteen infants^1^ were tested but excluded from data analysis because they did not complete the experiment (*n* = 12), or meet the EEG data quality standards (*n* = 7). Informed written consent was obtained from the infants’ legal guardians prior to data collection. This study was approved by the Ethics Committee of the School of Arts and Humanities of the University of Lisbon (13_CEI2019), and was carried out in compliance with the recommendations of the European Union Agency for Fundamental Rights and the Declaration of Helsinki.

**TABLE 1:** CSBS DP Checklist standard scores and percentile rank for the infants included in the study (*n* = 20, as two participants had missing data).

CSBS DP	Mean	SD	Range	Cut-off level for concern
Standard Score	97.00	8.73	82–117	< 81
Percentile Rank	42.50	20.09	12–87	≤ 10

### 2.2 Stimuli

**FIGURE 1:**
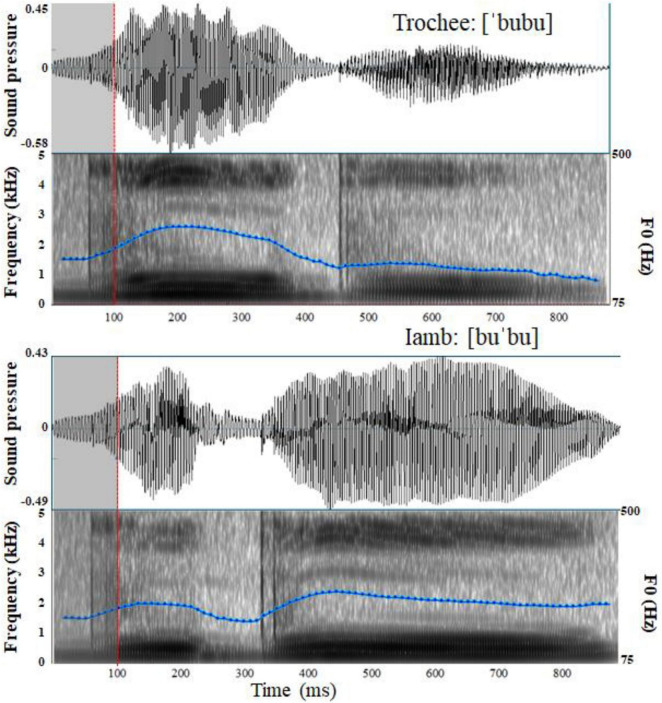
Waveforms and Spectrograms with fundamental frequency (F0) contour of the trochaic and iambic stress patterns.

The stimuli used in the current study were the same as those in Lu et al. (2018, 2023). In order to avoid changes in vowel quality due to vowel reduction in unstressed position, only high vowels ([i] and [u]) could be used since they do not show vowel reduction. Moreover, the presence of [i] in word final unstressed syllables is uncommon in the language, whereas the presence of [u] is extremely frequent. Therefore, the vowel [u] was used. In addition, the bilabial plosive was chosen, a consonant commonly used in ERP stress discrimination studies with infants (e.g., Weber et al., 2004; Friederici et al., 2007). The sequence [bubu], although not an actual word in the language, closely resembles words and word-like sequences commonly used in infant direct speech, like [ku'ku] ‘peekaboo’ which is one of the items of the EP CDI Short form for infants (Frota et al., 2016), and ['kuku] ‘cuckoo’. A female native EP speaker naturally produced the disyllable [bubu] with either a trochaic or an iambic stress pattern. Each stress pattern was produced twice, yielding a total of four tokens (['bubu]_1_, ['bubu]_2_, [bu'bu]_1_, and [bu'bu]_2_). All stimuli were nonsense words in EP and had a sampling rate of 22050 Hz. The average durations for the trochaic and iambic tokens were 872 milliseconds and 873 milliseconds respectively. Table 2 describes the total duration, as well as the duration, intensity and pitch for each of the two syllables for each token. Figure 1 presents illustrative waveforms and spectrograms with fundamental frequency (F0) contour of the trochaic and iambic tokens. To control the onset acoustic differences between tokens, we followed Weber et al. (2004) and substituted the first 100 milliseconds of ['bubu]_1_, ['bubu]_2_ and [bu'bu]_2_ with the first 100 milliseconds of [bu'bu]_1_. There was no discernible pitch discontinuity in any of the tokens after the manipulation, and physical differences between them began at 100 milliseconds. Three native EP speakers who did not participate in the experiment assessed all the stimuli to be perceptually natural.

**TABLE 2:** Duration, mean intensity, and mean pitch of the first_(1)_ and second_(2)_ syllable for each stimulus.

	Total Duration (ms)	Duration_1_ (ms)	Duration_2_ (ms)	Mean intensity_1_ (dB)	Mean intensity_2_ (dB)	Mean pitch_1_ (Hz)	Mean pitch_2_ (Hz)
['bubu]_1_	875	450	425	73	64	247	173
['bubu]_2_	868	435	433	72	68	245	181
[bu'bu]_1_	865	317	548	68	71	219	249
[bu'bu]_2_	880	313	567	71	70	222	246

### 2.3 Procedure

The experiment was carried out in a shielded and soundproof booth. Infants sat on their parents’ laps and were entertained by watching a silent animation video while the stimuli were delivered at a constant and comfortable volume through a loudspeaker. The video was playing during the full length of the EEG task and was the same for all infants. It consisted of an infant age-appropriate video showing slowly moving animated images. Infants heard two types of blocks that were generated in a passive oddball paradigm. In trochaic block, the trochaic tokens functioned as deviants and infrequently interrupted the regularly recurring iambic tokens. In iambic block, the iambic tokens behaved as deviants, and the trochaic tokens acted as standards. There were a total of 600 trials in each block (2 tokens × 50 + 2 tokens × 250), with each deviant token being presented 50 times and each standard token occurring 250 times. The stimulus presentation was pseudo-randomized, with each deviant following two to seven standards. From each block we selected a hundred clean standards (2 tokens × 50) that were not immediately preceding or after a deviant to compare with the same stress pattern delivered as deviants in the other block. We arbitrarily varied the offset-to-onset inter-stimulus interval between 800, 825, and 850 milliseconds to avoid participants’ anticipation of stimulus onset. To prevent participants’ fatigue, we split each block into two sub-blocks, resulting in four 8-min sub-blocks in total. The four sub-blocks were delivered through E-Prime 2.0 software (Psychology Software Tools, Pittsburgh, PA) in counterbalanced order across participants (Schneider et al., 2012). The entire experiment took 1 to 1.5 hours including preparation time and inter-block breaks.

### 2.4 EEG recording and averaging

The EEG signals were collected in DC mode using 32 Ag/AgCI electrodes at a sampling rate of 1000Hz. The electrodes were placed in an elastic cap (Quik-cap, Compumedics, NeuroScan, Victoria, Australia) according to the international 10–20 system and were connected to a SynAmps RT 64-channel Amplifier (Compumedics NeuroScan, Victoria, Australia). The ground electrode was defaulted by the EEG amplifier and the reference electrode was placed at the infants’ left mastoid. Two electrodes were positioned above and below the left eye of the infants to track their eye movements. All electrodes had an impedance of less than 10 kΩ.

EEGLAB (Delorme and Makeig, 2004) and ERPLAB (Lopez-Calderon and Luck, 2014) which are toolboxes in MATLAB (The MathWorks Inc.) were used to preprocess the EEG data. The EEG signals were band-pass filtered with 0.1–30 Hz^2^ and were re-referenced to average reference. Independent Component Analysis (ICA, EEGLAB) was applied to remove artifacts, such as eye movements, blinks, and muscle artifact. The continuous EEG was segmented into epochs from 200 milliseconds pre-stimulus to 800 milliseconds post-stimulus, corrected to a 200 milliseconds pre-stimulus baseline. Trials with voltage deviations more than ± 150μV on any electrode were discarded. Across participants, an average of 48 trials (SD = 17.28) for each stimulus type was retained for further analysis. ERPs were averaged separately for each participant, each electrode, and each stimulus type. Difference waves were obtained for the trochaic and iambic stress patterns respectively by subtracting the ERP evoked by the clean standards from the ERP evoked by the corresponding deviants.

### 2.5 Data analysis

According to previous ERP studies on infant stress perception (e.g., Friedrich et al., 2009), we calculated the mean amplitudes of five consecutive time windows of 100 milliseconds from 200 to 700 milliseconds after the stimulus onset. The mean amplitudes were statistically analyzed into four regions of interest (ROIs): left-frontal (LF: F7, F3, FT7, and FC3), right-frontal (RF: F4, F8, FC4, and FT8), left-posterior (LP: TP7, CP3, P7, and P3), and right-posterior (RP: CP4, TP8, P4, and P8). A series of 2 × 2 × 2 repeated measure ANOVAs were performed on the mean amplitudes in the four ROIs for each stress pattern, with Discrimination (deviant vs. standard), Anteriority (anterior vs. posterior), and Hemisphere (left vs. right) as within-subject factors. For each stress pattern, we also performed 2 × 5 repeated measure ANOVAs on midline electrodes with the factors of Discrimination (deviant vs. standard) and Site (Fz, FCz, Cz, CPz, and Pz). The Greenhouse-Geisser correction was applied to all *F*-values and *p*-values and the Bonferroni correction was used for multiple comparisons.

## 3 Results

**FIGURE 2:**
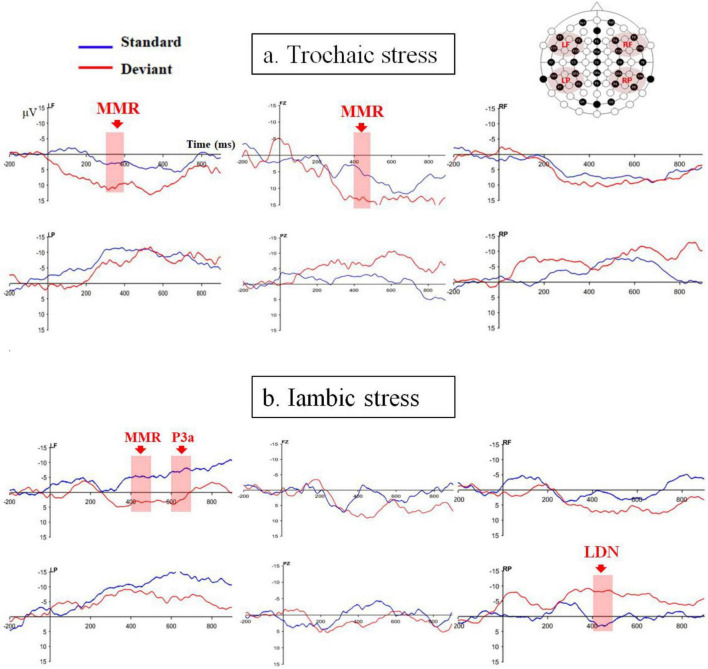
ERPs averaged for the four ROIs as well as the two midline electrodes (Fz and Pz) for **(a)** trochaic and **(b)** iambic stress patterns.

Figure 2 displays the ERPs averaged for the four analyzed ROIs as well as the two midline electrodes (Fz and Pz) for the trochaic and iambic stress patterns.

### 3.1 Trochaic stress

For trochaic stress, neither the main effect of Discrimination nor the main effect of Hemisphere was significant in any of the time windows. The main effect of Anteriority was significant in all five time windows {200–300ms: [*F* (1, 21) = 19.84, *p* < 0.001, η^2^ = 0.49]; 300–400ms: [*F* (1, 21) = 31.18, *p* < 0.001, η^2^ = 0.60]; 400–500 ms: [*F* (1, 21) = 39.51, *p* < 0.001, η^2^ = 0.65]; 500–600 ms: [*F* (1, 21) = 31.85, *p* < 0.001, η^2^ = 0.60]; 600–700 ms: [*F* (1, 21) = 46.87, *p* < 0.001, η^2^ = 0.69]}. The mean amplitudes were positive in the frontal electrodes, while were negative in the posterior electrodes. In the time window of 300–400ms, the interaction of Discrimination × hemisphere was significant [*F* (1, 21) = 4.44, *p* = 0.047, η^2^ = 0.17]. There was a significant discrimination effect in the left hemisphere [*t* (21) = −2.31, *p* = 0.031], but not in the right hemisphere [*t* (21) = 0.36, *p* = 0.72]. No other significant interactions were found.

The analysis on the midline electrodes showed that the main effect of Site reached significance in the time windows of 200–300ms: [*F* (2, 39) = 6.34, *p* = 0.005, η^2^ = 0.23], 300–400 ms [*F* (2, 32) = 10.06, *p* = 0.001, η^2^ = 0.32], 400–500 ms [*F* (2, 35) = 11.45, *p* < 0.001, η^2^ = 0.35], 500–600 ms [*F* (2, 38) = 12.62, *p* < 0.001, η^2^ = 0.38], and 600–700 ms [*F* (2, 40) = 19.24, *p* < 0.001, η^2^ = 0.48]. Moreover, there were significant interactions of Discrimination × Site in the time windows of 300–400 ms [*F* (2, 40) = 3.86, *p* = 0.031, η^2^ = 0.16], and 400–500 ms [*F* (2, 49) = 3.62, *p* = 0.028, η^2^ = 0.15]. *Post hoc* analyses revealed a significant discrimination effect at electrode Fz with a positive polarity in the time window of 400–500 ms [*t* (21) = −2.41, *p* = 0.025]. No other significant discrimination effect was found.

### 3.2 Iambic stress

For iambic stress, the main effect of Anteriority was also significant in all five time windows {200–300 ms: [*F* (1, 21) = 4.91, *p* = 0.038, η^2^ = 0.19]; 300–400 ms: [*F* (1, 21) = 9.50, *p* = 0.006, η^2^ = 0.31]; 400–500 ms: [*F* (1, 21) = 8.66, *p* = 0.008, η^2^ = 0.29]; 500–600 ms: [*F* (1, 21) = 10.42, *p* = 0.004, η^2^ = 0.33]; and 600–700 ms: [*F* (1, 21) = 11.08, *p* = 0.003, η^2^ = 0.35]}. The mean amplitudes were positive in the frontal regions, and were negative in the parietal regions. In addition, in the time windows of 400–500 ms [*F* (1, 21) = 10.89, *p* = 0.003, η^2^ = 0.34], 500–600 ms [*F* (1, 21) = 7.24, *p* = 0.014, η^2^ = 0.26], and 600–700 ms [*F* (1, 21) = 6.17, *p* = 0.021, η^2^ = 0.23], there was a main effect of Hemisphere. In the time window of 300–400 ms, the interaction of Discrimination × Hemisphere reached significance [*F* (1, 21) = 7.05, *p* = 0.015, η^2^ = 0.25]. However, further analysis demonstrated that the discrimination effect was borderline at the left hemisphere [*t* (21) = −2.00, *p* = 0.058] and not significant at the right hemisphere [*t* (21) = 1.15, *p* = 0.26]. In the time window of 400–500 ms, the interaction of Discrimination × Anteriority [*F* (1, 21) = 5.19, *p* = 0.033, η^2^ = 0.20] and the interaction of Discrimination × Hemisphere × Anteriority [*F* (1, 21) = 10.19, *p* = 0.004, η^2^ = 0.33] were significant. The LF region exhibited a significant discrimination effect with positive polarity [*t* (21) = −2.88, *p* = 0.009], and the RP region showed a significant discrimination effect with negative polarity [*t* (21) = 3.26, *p* = 0.004]. There was also a significant interaction of Discrimination × Hemisphere × Anteriority in the time window of 600 – 700ms [*F* (1, 21) = 5.51, *p* = 0.029, η^2^ = 0.21]. Further analysis only revealed a significant positive-going discrimination effect in the LF region [*t* (21) = −3.11, *p* = 0.005].

The analysis on the midline electrodes only exhibited a significant main effect of Site in the time window of 600–700 ms [*F* (2, 45) = 3.49, *p* = 0.036, η^2^ = 0.14]. No additional main effect or interaction was discovered in any other time windows.

### 3.3 Difference waves

**FIGURE 3:**
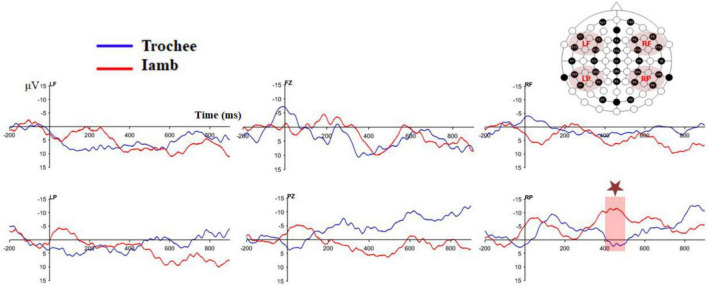
Difference waves of the four ROIs as well as the two midline electrodes (Fz and Pz) for the trochaic and iambic stress patterns.

**FIGURE 4:**
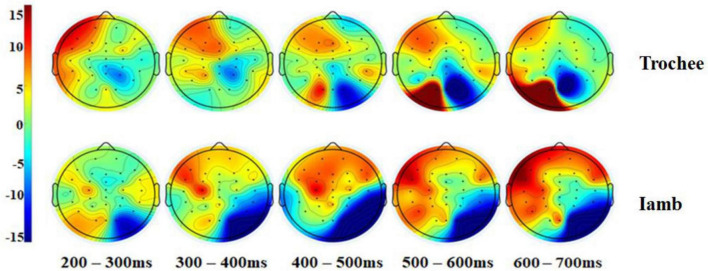
Topographic maps for the trochaic and iambic stress patterns in the five time windows.

Figure 3 displays the difference waves of the four ROIs and the two midline electrodes (Fz and Pz) for the trochaic and iambic stress patterns. Figure 4 illustrates the topographic maps for the trochaic and iambic stress patterns in the five time windows.

Näätänen et al. (2019) have pointed out that because of the low signal-to-noise ratio and instability of infant data, it is essential to make sure that the responses under examination differ significantly from zero. Therefore, we performed the following tests to double check the discrimination effects we found in the previous section. The mean amplitudes of the LF electrodes for the trochaic difference wave were significantly different from zero in the time windows of 200–300 ms [*t* (21) = 2.38, *p* = 0.027], and 300–400 ms [*t* (21) = 2.25, *p* = 0.036]. Moreover, the amplitude of the electrode Fz significantly differed from zero in the time window of 400−500 ms [*t* (21) = 2.41, *p* = 0.025].

For the iambic difference wave, the mean amplitudes of the LF electrodes were significantly different from zero in the time windows of 300−400 ms [*t* (21) = 2.40, *p* = 0.026], 400−500 ms [*t* (21) = 2.88, *p* = 0.009], 500−600 ms [*t* (21) = 3.16, *p* = 0.005], and 600−700 ms [*t* (21) = 3.11, *p* = 0.005]. In addition, the mean amplitudes of the RP electrodes were significantly different from zero in the time windows of 300−400 ms [*t* (21) = −2.41, *p* = 0.025] and 400−500 ms [*t* (21) = −3.26, *p* = 0.004]. As in the trochaic condition, the amplitude of the electrode Fz also significantly differed from zero in the time window of 400−500 ms [*t* (21) = 2.46, *p* = 0.023].

We performed a series of 2 × 2 × 2 repeated measures ANOVAs on the difference waves in the five time windows to directly compare the differences between the trochaic and iambic stress. The result showed a significant interaction of Stress × Hemisphere × Anteriority in the 400−500 ms [*F* (1, 21) = 5.31, *p* = 0.031, η^2^ = 0.20], and 500−600ms [*F* (1, 21) = 5.18, *p* = 0.033, η^2^ = 0.20] time windows. Post hoc analysis exhibited a significant difference between trochaic and iambic stress in the RP region in the 400−500ms time window [*t* (21) = 2.25, *p* = 0.035], with iambic stress displaying a prominent negativity [iambic RP vs. 0: *t* (21) = −3.25, *p* = 0.004].

The amplitudes of the midline electrodes were also submitted to five 2 × 5 repeated measure ANOVAs, with stress (trochee vs. iamb), and Site (Fz, FCz, Cz, CPz, and Pz) as within-subject factors. The main effect of Site was significant in the 400−500 ms time window [*F* (2, 40) = 3.51, *p* = 0.041, η^2^ = 0.14]. There was no additional significant main effect or interaction in any other time windows. Table 3 summarizes all the significant results in the five time windows.

**TABLE 3:** Main effects and interactions in the five time windows for (a) trochaic stress, (b) iambic stress and (c) difference waves.

	Time windows				
	200–300	300–400	400–500	500–600	600–700
**a. Trochaic stress**					
Anteriority (Ant)	***	***	***	***	***
Discrimination (Dis) x Hemisphere (Hem)		*			
Dis−Fz			*		
**a. Iambic stress**					
Ant	*	**	**	**	**
Hem			**	*	*
Dis x Hem		*			
Dis x Ant			*		
Dis x Hem x Ant			**		*
**c. Difference waves**					
Stress x Hem x Ant			*	*	

***p ≤ 0.001,

**p ≤ 0.01,

*p < 0.05.

## 4 Discussion

The current study used event-related potentials (ERPs) to examine EP-learning infants’ stress discrimination abilities in the absence of vowel reduction. We found that both the trochaic and iambic stress patterns elicited a p-MMR. For trochaic stress, the p-MMR was evident in the left-frontal region in the 300–400ms time window, and was also significant at Fz in the 400–500ms time window. For iambic stress, a salient p-MMR was detected in the left-frontal area in the 400–500ms time window. In addition, iambic stress displayed a prominent negativity in the right-parietal region in the 400–500 ms time window, and another positive component in the left-frontal area in the time window of 600–700 ms. Our findings are compatible with Frota et al. (2020) study that used eye-tracking to demonstrate that 5–6 month-old EP-learning infants were sensitive to the trochaic/iambic stress contrast. However, Frota et al. did not find reciprocal discrimination of stress patterns, but reported an asymmetrical perception or preference for iambic stress. Thus, the present findings add to previous results by showing that early stress perception is characterized by (i) the discrimination of both trochaic and iambic patterns, and (ii) a language-specific processing advantage for iambic stress.

Vowel reduction has been regarded as the main cue for EP stress discrimination. Previous behavioral studies have demonstrated that in the absence of vowel reduction EP adult speakers demonstrated a “stress deafness” effect comparable to that observed in speakers of languages with no lexical stress or fixed stress (Correia et al., 2015; Lu et al., 2018). However, in an ERP study, EP adult speakers displayed MMN responses in both the trochaic and iambic conditions, suggesting that without vowel reduction they could inattentively discriminate both stress patterns (Lu et al., 2018). In the present study, 5–7 month-old EP-learning infants exhibited similar results as the EP adult speakers, manifested by prominent p-MMRs in both the trochaic and iambic conditions. P-MMRs have been observed in newborns and infants for the discrimination of changes in various features, such as in segmental phonetic contrasts (e.g., Dehaene-Lambertz and Dehaene, 1994; Dehaene-Lambertz and Pena, 2001), frequency of pure tones (Leppänen et al., 1997; Morr et al., 2002), and vowel duration (Friederici et al., 2002). Studies measuring MMRs across age have reported that adult-like MMN may emerge between 4 and 6 months of age, and as children get older it becomes more prevalent (e.g., He et al., 2009; Trainor et al., 2003). Other research has suggested that p-MMR is likely to be elicited by small deviants while MMN tends to be induced by large deviants (e.g., Cheng et al., 2015; Maurer et al., 2003; Morr et al., 2002). For instance, Morr et al. (2002) found that infants younger than 12 months revealed a p-MMR in response to a smaller pure tone deviant, whereas infants as young as 2 months showed an adult-like MMN for a larger deviant. These results suggest that the polarity of the MMR may depend on stimuli-related factors as well as infants’ maturational status (Cheng and Lee, 2018). Although its exact neural function has been debated, the MMR has been claimed to represent auditory discrimination irrespective of its polarity (see Fitzgerald and Todd, 2020, for a review). Therefore, our findings indicate that, at the pre-attentive stage, 5–7-month-old EP-learning infants already show stress discrimination of both trochaic and iambic patterns, like EP adult speakers.

In addition to the p-MMR, we found a prominent right-parietal negativity in the 300−500 ms time windows, and a salient left-frontal positivity in the 600−700 ms time window for the iambic condition. Although the negative component emerges in the same time window as the LDN reported in previous studies (e.g., Korpilahti et al., 2001), it has a different scalp distribution from the LDN. Mueller et al. (2008) also found a parietal LDN component overlapping with the frontal MMN in young children, suggesting that the scalp distribution of LDN in children may be different from that in adults. In the present study, the LDN-like component occurred in similar time windows as the p-MMR for the iambic stress, probably reflecting deviance rule extraction (e.g., Zachau et al., 2005), or an increase in involuntary shifting (e.g., Shestakova et al., 2003). Researchers have not yet reached an agreement regarding the role of LDN, but studies seemed to suggest that the presence of LDN might be language-specific and the function of LDN might differ across participant characteristics (e.g., Yu et al., 2017) and various tasks (e.g., Shestakova et al., 2003).

The significant left-frontal positivity in the 600–700 ms time window could be regarded as a P3a component, since some studies have indicated that children typically exhibit a longer P3 component latency than adults (e.g., Cycowicz et al., 1996; Fuchigami et al., 1995). Previous studies on stress processing by adult speakers have reported a late positive component (LPC) and found a strong correlation between LPC amplitude and recognition memory (e.g., Böcker et al., 1999; Broś et al., 2021). Because of this correlation researchers claimed that the LPC might be a late instance of P3, manifesting categorization or context updating (Donchin et al., 1978). Besides, previous longitudinal studies have reported contradictory results regarding the developmental trajectory of the P3a magnitude. Some studies found that the magnitude of P3a increases and matures toward adult-like positivity until late adolescence (e.g., Linnavalli et al., 2018; Putkinen et al., 2014), while others showed that the P3a amplitude decreases with age (e.g., Gumenyuk et al., 2004; Mahajan and McArthur, 2015). Linnavalli et al. (2022) speculated that the decrease of P3a magnitude might be attributed to adults’ more effective suppression of involuntary attention.

In the present study, the prominent P3a together with the LDN component seemed to indicate that the EP-learning infants perceived lexical stress asymmetrically, with more robust perception of iambic stress. This co-occurring MMR-P3a-LDN complex has been reported in previous research using linguistic stimuli, and has been claimed to reflect further auditory discriminative processing or even complex cognitive processes (e.g., Azaiez et al., 2023; Jakoby et al., 2011). Our results are also consistent with previous behavioral and ERPs findings on EP adult speakers (Lu et al., 2018), as well as findings from an eye-tracking study with 5–6 month-old EP-learning infants (Frota et al., 2020). For adults, the behavioral data from a discrimination task (ABX) revealed an iambic advantage, with more accurate and fast responses when X had iambic stress. In addition, the MMN and late negative components in the iambic condition were more negative and spanned over a larger temporal window. For infants, the eye-tracking data showed longer looking times for iambic stress indicating that infants preferred this stress pattern. The processing advantage for the iambic stress pattern could be explained by a language-specific combination of features, as argued in Frota et al. (2020), in particular, the high weight of duration as a perceptual correlate for stress, along with the phonological patterns that influence the frequency distribution of stress patterns in EP. Several studies have suggested that trochaic and iambic groupings rely heavily on acoustic cues, emerging from prominence marked by pitch and intensity in the case of trochaic patterns and prominence marked by duration in the case of iambic patterns (Bion et al., 2011; Peña et al., 2016). In the present study, duration was the primary correlate for stress, and thus we would expect an iambic advantage. Infants’ perception of stress has also been proposed to rely on the language-specific frequency distribution of stress patterns. Studies have shown that infants’ early stress preferences or asymmetrical stress perception tend to match the predominant stress pattern in their native language (Jusczyk et al., 1993; Segal and Kishon-Rabin, 2012; Weber et al., 2004). In EP, iambic stress predominates if frequency is computed beyond lexical words and the phonetics and phonology of monosyllables and cliticization are taken into account (Vigário et al., 2010). The iambic advantage found in the present study indicates that we should consider the phonetic and phonological features of the language in connected speech when computing the language-specific stress frequency. The present findings thus confirmed that by 5–7 months EP-learning infants already developed a language-specific asymmetrical discrimination towards iambic stress at the pre-attentive stage, echoing earlier findings in Frota et al. (2020) which showed that EP-learning infants’ demonstrated an iambic preference at the attentive stage.

In conclusion, the present study was the first to use the ERPs method to investigate stress processing by EP-learning infants. Our results indicated that both the trochaic and iambic conditions elicited a p-MMR, showing that in the absence of vowel reduction EP-learning infants at 5–7 months could pre-attentively discriminate stress patterns. In addition, the iambic condition revealed a prominent LDN-like component, as well as a P3a component, suggesting a processing advantage for iambic stress. Thus, our findings present the first evidence for reciprocal discrimination of stress patterns in EP-learning infants, showing that stress processing might also differ at the pre-attentive and attentive stages in infants. Moreover, in line with earlier results for adults and infants, our findings provide further support for a processing advantage for iambic stress in EP, found in both the attentive and pre-attentive stages. This suggests that EP-learning infants seem to develop their stress perception abilities through an asymmetrical perception mechanism triggered by iambic stress. These findings underline the need to study developing stress processing in languages with different combinations of stress-related phonetic and phonological properties, advancing the understanding of the language-specific factors that influence the acquisition of stress.

## Data Availability

The raw data supporting the conclusions of this article will be made available by the authors, without undue reservation.

## Footnotes

1 If an infant cried inconsolably, the session was terminated; if an infant’s data failed to reach the minimum 25 of clean trials for each experimental condition, his or her data were dropped from analysis. Stets et al. (2012) examined 149 ERP articles published between 1999 and 2010 and concluded that compared with other infant study designs EEG seems to have particularly high attrition rates, with an average of 49.2 percent.

2 We have also processed the data using a 1–30 Hz band-pass filter. No significant discrimination effect was observed for the trochaic stress pattern, while a positive discrimination response (MMR) at 500–600 ms after stimulus onset was found for the iambic stress pattern. Some studies have found that the low-pass filtering cutoff has minimal impact on the detectability of MMN or MMR, whereas a higher high-pass filter cutoff may significantly reduce the amplitude of the waking MMN (e.g., Sabri and Campbell, 2002; Uhler et al., 2018). Besides, Luck (2022) has pointed out that the 0.1 Hz cutoff for the high-pass filter works best in most cases. Therefore, we used the 0.1–30 Hz band-pass filter. It is worth noting that no matter whether the 0.1–30 Hz or the 1–30 Hz bandpass filter was utilized, the result revealed a processing advantage for the iambic stress pattern.
